# Associations between air pollution and perceived stress: the Veterans Administration Normative Aging Study

**DOI:** 10.1186/1476-069X-14-10

**Published:** 2015-01-27

**Authors:** Amar J Mehta, Laura D Kubzansky, Brent A Coull, Itai Kloog, Petros Koutrakis, David Sparrow, Avron Spiro, Pantel Vokonas, Joel Schwartz

**Affiliations:** Department of Environmental Health, Harvard School of Public Health, Landmark Ctr, West 415, 401 Park Dr, Boston, MA 02215 USA; Department of Social and Behavioral Sciences, Harvard School of Public Health, Boston, USA; Department of Biostatistics, Harvard School of Public Health, Boston, USA; Department of Geography and Environmental Development, Ben-Gurion University of the Negev, Beer Sheva, Israel; The VA Normative Aging Study, VA Boston Healthcare System, Boston, USA; The Channing Laboratory, Brigham and Women’s Hospital, Harvard Medical School, Boston, USA; Department of Medicine, Boston University School of Medicine, Boston, USA; Department of Epidemiology, Boston University School of Public Health, Boston, USA; Department of Psychiatry, Boston University School of Medicine, Boston, USA

**Keywords:** Aged, Air pollution, Male, Particulate matter, Prospective studies, Stress, Psychological

## Abstract

**Background:**

There is mixed evidence suggesting that air pollution may be associated with increased risk of developing psychiatric disorders. We aimed to investigate the association between air pollution and non-specific perceived stress, often a precursor to development of affective psychiatric disorders.

**Methods:**

This longitudinal analysis consisted of 987 older men participating in at least one visit for the Veterans Administration Normative Aging Study between 1995 and 2007 (n = 2,244 visits). At each visit, participants were administered the 14-item Perceived Stress Scale (PSS), which quantifies stress experienced in the previous week. Scores ranged from 0–56 with higher scores indicating increased stress. Differences in PSS score per interquartile range increase in moving average (1, 2, and 4-weeks) of air pollution exposures were estimated using linear mixed-effects regression after adjustment for age, race, education, physical activity, anti-depressant medication use, seasonality, meteorology, and day of week. We also evaluated effect modification by season (April-September and March-October for warm and cold season, respectively).

**Results:**

Fine particles (PM_2.5_), black carbon (BC), nitrogen dioxide, and particle number counts (PNC) at moving averages of 1, 2, and 4-weeks were associated with higher perceived stress ratings. The strongest associations were observed for PNC; for example, a 15,997 counts/cm^3^ interquartile range increase in 1-week average PNC was associated with a 3.2 point (95%CI: 2.1-4.3) increase in PSS score. Season modified the associations for specific pollutants; higher PSS scores in association with PM_2.5_, BC, and sulfate were observed mainly in colder months.

**Conclusions:**

Air pollution was associated with higher levels of perceived stress in this sample of older men, particularly in colder months for specific pollutants.

**Electronic supplementary material:**

The online version of this article (doi:10.1186/1476-069X-14-10) contains supplementary material, which is available to authorized users.

## Background

There is emerging experimental and observational evidence from recent studies to suggest that exposure to ambient air pollution may be associated with neurobehavioral outcomes. Findings from experimental studies in mice suggest that air pollution may be associated with enhanced bias towards immediate reward [1] and depression-like responses [2, 3]. An observational study of children born to non-smoking mothers observed an association between pre-natal ambient exposure to polyaromatic hydrocarbons and symptoms of anxiety and/or depression at ages between six and seven years [4]. Observational studies in adults have also demonstrated associations between air pollution and depression [5, 6], and suicide [7, 8]. Similar findings from observational studies are also shown for indoor air pollutants resulting from secondhand smoke [9, 10] and biomass fuel [11]. Associations between air pollution and mood [12] and depressive [13] symptoms, and psychiatric emergencies [14] have also been shown in early observational studies.

Less known are the potential effects of air pollution on psychological stress, and more specifically perceived stress, which when it occurs as a chronic state is thought to contribute to the development of affective psychiatric disorders, including depression [15–17]. Perceived stress has been linked with increased likelihood of biological dysregulation including inflammation [18, 19], and greater risk of cardiovascular disease and premature mortality [20–23]. Both social and physical determinants (e.g., socioeconomic status, noise, crowding), of perceived stress have been evaluated [24–26], and it is also hypothesized from earlier studies that the association between air pollution and depression may be mediated by perception of air quality [12, 27]. However, whether objectively measured air pollution is directly associated with psychological stress in settings with relatively low-levels of ambient air pollution, where perceived air quality is likely to have minimal influence, is unknown.

For this study, we evaluated whether air pollution levels, averaged over of one and up to the previous four weeks, were associated with non-specific perceived stress in a cohort of older community-dwelling men living in the Boston Metropolitan Area. Taking consideration of prior work suggesting that air pollution is associated with activation of the hypothalamic-pituitary-adrenal axis [28] and hippocampal cytokine inflammation [3], we hypothesized that objectively measured air pollution is associated with higher perceived stress as mediated by inflammation and glucocorticoid activity. We also account for a range of potential confounders including age, individual level indicators of socioeconomic status, physical activity, anti-depressant medication use, seasonality, and meteorology. Evaluating the relationship between air pollution and perceived stress in this sample of community-dwelling individuals may provide insight into one mechanism by which exposure to air pollution may be associated with risk of affective psychiatric disorders.

## Methods

### Study population and design

Participants included in this analysis were enrolled in the Veterans Administration Normative Aging Study (NAS), an ongoing longitudinal study of aging established in 1963, details of which have been published previously [29]. Briefly, the NAS is a closed cohort of 2,280 male volunteers from the Greater Boston area aged 21–81 years at entry, who enrolled after an initial health screening determined that they were free of known chronic medical conditions. The present study was approved by the Human Research Committees of the Harvard School of Public Health, and the Department of Veterans Affairs Boston Healthcare System, and written informed consent was obtained from participants prior to participation. The men have been reevaluated every 3–5 years by using detailed on-site physical examinations and questionnaires. Dropout has been less than 1% per year in this cohort and predominantly occurs when participants move out of the study area. The other major reason for loss to follow-up has been mortality.

Eligibility for the current study required continued participation as of 1995 when air pollution monitoring began. At this time, 1,118 individuals remained in the study, participating in at least one study visit between 1995 and 2007 (Additional file 1: Figure S1). The NAS study population is predominantly white, and 2% of the study population is black. The current analysis was restricted to 987 participants with complete information on the outcome, exposures, and covariates of interest in at least one visit. In comparison with the 131 eligible NAS participants who were not included in this analysis, the 987 included participants were on average significantly younger, more educated, and less likely to be current smokers (Additional file 1: Table S1). This analysis included a maximum of 2,244 visits from 987 participants who had at least one but ranging up to four scheduled visits over the course of the study; the number of participant-visits utilized in the analysis varied by air pollutant of interest as we excluded participant-visits for which air pollution data in the relevant time period was not available. Refer to Figure S1 (Additional file 1) outlining the inclusion of participants for this analysis. For the purpose of this analysis, we will refer to the first visit during the specified study period as the baseline visit.

### Assessment of perceived stress

The 14-item Perceived Stress Scale (PSS) [15], a validated measure of stress appraisal, was used to ascertain the degree to which respondents felt their lives were “unpredictable, uncontrollable, and overloaded” during the previous week. Each item was scored on a 5-point scale that ranges from “never” (0) to “very often” (4). The scores for the positive items in the 14-item scale were reversed, and a total score was obtained by summing all items with scores ranging from 0 to 56, so that higher scores indicate higher levels of perceived stress. The PSS is a widely used stress appraisal measure; it correlates strongly with other measures of psychosocial stress (e.g., life events) with depressive and physical symptomatology, and has been shown to be associated with greater risk of poor health [15]. Consistent with previous studies [30–32], PSS scores were modestly stable across visits in this sample (Intraclass correlation coefficient = 0.32). In this study sample, the PSS score approximated normal distribution and was characterized as a continuous variable.

### Assessment of air pollution and meteorology

Ambient particulate pollutant concentrations were monitored at our Harvard Air Pollution Supersite located near downtown Boston 1 km from the VA medical center. Particle measurements included ambient particulate matter ≤2.5 μm in diameter (PM_2.5_), black carbon (BC), particle number counts (PNC), and sulfate particles (SO_4_^2-^). We measured hourly PM_2.5_ concentrations with a Tapered Element Oscillation Microbalance (Model 1400A, Rupprecht and Pastashnick, East Greenbush, NY), and BC concentrations using an Aethalometer (Magee Scientific Co., Model AE-16, Berkeley, CA). BC is associated with traffic emissions especially those related to diesel fuel combustion. Missing hourly concentration data for PM_2.5_and BC were imputed using regression modeling, including a long term time trend, day of week, hour of day, temperature, relative humidity, barometric pressure and nitrogen dioxide concentrations (NO_2_) as predictors. We measured hourly PNC (0.007 – 3 μm particles per cm^3^) with a Condensation Particle Counter (TSI Inc, Model 3022A, Shoreview, MN). Particle number is mostly influenced by freshly generated particles from local traffic [33]. We determined daily SO_4_^2-^ concentrations with a Sulfate Particulate Analyzer (Thermo. Electron Co., Model 5020, Franklin, MA) from 1999 to 2003. Subsequently, SO_4_^2-^ levels were calculated from elemental sulfur, measured by X-Ray Fluorescence from particle filters. For the year of overlap we fit a calibration regression, which had an R^2^ over 0.9, and a slope of 1. SO_4_^2-^ particles are formed through the oxidation of sulfur dioxide emitted primarily by coal- and oil-burning power plants and can be transported regionally over long distances (e.g., hundreds of kilometers) [34]. We obtained hourly O_3_ and NO_2_ concentration data (ppm) from local state monitors within the Greater Boston, and concentrations were estimated by averaging data from all of the available sites. The median of the mean distances of the participant homes from the central site monitoring station was 20.7 km (Range: 0.9, 144.2); the medians of the mean distances of the participant homes from the O_3_, and NO_2_ monitors were 24.9 km (Range: 8.4, 128.3), and 23.5 km (Range: 5.7, 127.1), respectively.

Hourly ambient temperature and dew point temperature data were obtained from the first order National Weather Service station at Boston Logan airport (8 km from the medical center). We calculated apparent temperature, a human discomfort index [35], as: apparent temperature = -2.653 + (0.994 × ambient temperature) + (0.0153 × (dew point temperature)^2^ ), where ambient and dew point temperature are measured in Celsius. Pollutant sampling, processing of samples, analysis and reporting were conducted according to standard operating procedures [36]. For each air pollutant that was measured from stationary monitors, we considered the medium-term exposure windows of 1, 2, and 4 week moving average preceding each participant’s examination.

Additionally, all home addresses of participants in the VA Normative Aging Study were geocoded, and predicted average exposures to BC and PM_2.5_ were estimated using validated models described previously [37, 38] by averaging the daily predicted estimates at the participant’s residential address or addresses for the 1, 2, and 4 weeks before each clinical visit. More specifically, predicted BC was estimated from a nonlinear land-use regression model which was applied to within the greater Boston metropolitan area [36], and predicted PM_2.5_ exposure was derived from satellite aerosol optical depth measurements to generate both exposure to PM_2.5_ at the area level (10 × 10 km) and the local level (100 m) based on local land use variables [38]. Refer to Additional file 1 for more detailed description of the models predicting exposure to BC and PM_2.5_.

### Assessment of other covariates

At each examination, participants were followed up by physical examination, updating of medical history, and measurement of biomarkers. Forced expiratory volume in one second (FEV_1_, ml) was measured from pulmonary function tests as previously reported and in accordance with American Thoracic Society standards [39, 40]. Weight and height were measured with participants wearing only socks and underpants, from which body mass index (BMI) (weight/height^2^) was calculated. Physical activity was assessed on a scale derived by Paffenbarger et al. [41]. Responses to questions about the number of flights of stairs climbed per day, walking pace, and frequency of various sports activities were used to derive a physical activity variable that assessed in metabolic equivalent tasks score (METs) per week. Maximum educational attainment, specifically years of education, was ascertained by questionnaire in early visits. At each visit, participants were also asked to bring their current medical prescriptions; antidepressant medication use included any of the following classes of anti-depressants: serotonin-norepinephrine-reuptake inhibitors, selective serotonin-reuptake inhibitors, serotonin modulators, norepinephrine-dopamine reuptake inhibitors, and noradrenergic and specific serotonergic anti-depressants, and tricyclics and other norepinephrine-reuptake inhibitors.

### Statistical analysis

All statistical analyses were carried out using SAS Version 9.2 (SAS Institute, Cary, NC). We used time-varying linear mixed-effects regression models with random participant-specific intercepts (via PROC MIXED), accounting for the correlation of repeated measures [42], to model PSS score as a continuous function of medium-term moving average exposure (1, 2, and 4 weeks prior to visit) of PM_2.5_, BC, PNC, SO_4_^2-^, NO_2_, and O_3_ as measured from the stationary monitoring sites. We also modeled PSS score as a continuous function of medium-term moving average exposure of predicted PM_2.5_ and BC as estimated from the spatio-temporal models, as we wished to evaluate whether the associations for predicted PM_2.5_ and BC were consistent with those exposures measured from the stationary monitoring site. Estimated associations between air pollution and perceived stress are given per interquartile range of the pollutant for the specific moving average.

We fit each moving average exposure of each pollutant individually at a time in an established covariate model that included potential determinants of perceived stress and potential confounders of the association between air pollution and perceived stress. These covariates included age at visit, race, years of education (<12, 12, 13–15, >15 as reference), 24-hour average apparent temperature (°C), seasonality (sine and cosine of calendar day), weekday of visit, physical activity at visit (METs per week, in quartiles or missing data, lowest quartile as reference), and use of anti-depressant medication at visit (no as reference). We did not consider additional adjustment for smoking status and alcohol consumption in our primary analysis, as these behavioral risks are potentially effects of perceived stress [43, 44]. However, we further adjusted for smoking status (current, former, never as reference) and alcohol consumption (≥2 drinks/day, < 2 drinks/day as reference) as a sensitivity analysis. We also excluded participant-visits with reported use of anti-depressant medication (n = 88) in all models as a sensitivity analysis.

Considering the epidemiologic evidence from community and population-based studies which have shown a seasonal influence on mood states and depressive symptoms [45, 46], we also hypothesized that seasonal variation may be an underlying susceptibility factor of higher stress ratings associated with air pollution. Thus, we evaluated if season (April-September, October-March for warm and cold season, respectively) modified the associations between air pollution and perceived stress, by testing for interaction between warm season (cold season as reference) and moving average air pollution exposure.

Healthier men may have be more likely to participate in a subsequent follow-up visits, so we used stabilized inverse probability weights (IPWs) to correct for this potential survival bias [47] in all models. We calculated the probability of participating in subsequent visits using two logistic regression models. The first logistic regression model calculated the probability of participating in the subsequent visit as a function of air pollution concentration measured at the previous visit. Consistent with methods previously described [48], the second logistic regression model calculated the probability of participation in the follow-up visit in a fully adjusted model including air pollution concentration, PSS score, age, body mass index (kg/m^2^), FEV_1_ (ml), race (black, white as reference), smoking status (current smoker, recent quitter, longtime quitter, never smoker as reference), cumulative pack years smoked, years of education (<12, 12, 13–15, >15 as reference), hypertension, total cholesterol (mg/dL), diabetes mellitus, and physician diagnosis of asthma, chronic bronchitis, and emphysema measured at the previous visit. Subsequently, we estimated stabilized IPWs for participation in the follow-up visit by taking the ratio of the probability estimated from the first logistic regression model over the probability estimated from the second logistic regression model. The purpose of fitting air pollution exposure in the logistic models is to prevent estimation of extreme values for the weights; all weighted models met the necessary condition for correct model specification such that the values for stabilized weights have a mean of one [47]. The stabilized inverse probability weight at the baseline visit was 1. As a sensitivity analysis, we estimated the inverse probability of being included in the analysis among eligible participants; rather than a constant of ‘1’, the inverse probability of being included in the analysis was assigned to the baseline visit.

## Results

Characteristics of all participants at the baseline study visit are summarized in Table 1. The mean age of all participants was approximately 69 years, and the majority of them (71%) participated in at least 2 visits. Less than three percent of all participants reported use of psychiatric medication, and 14 of these 27 participants reported specific use of a psychotropic medication. Across participants, mean PSS scores remained relatively stable at each visit, and generally, modest correlations were observed for PSS score between visits (*r* ≥ 0.3) (Table 2). However, weaker correlations were observed for PSS score between the first and third, and first and fourth visits (*r* = 0.2).

**Table 1:** **Characteristics of 987 participants at the baseline visit**

Characteristics	n (%) ^*^
Total number of visits	
One	288 (29)
Two	289 (29)
Three	262 (27)
Four	148 (15)
Age, mean (SD)	69.1 (7.0)
Race	
White	972 (98)
Black	15 (2)
Years of education, mean (SD)	14.8 (2.9)
Use of anti-depressant medication	27 (3)
Physical activity (METS/week), mean (SD)	17.0 (21.7)
Missing information on physical activity	25 (3)

*Abbreviations:* *SD* standard deviation, *METS* metabolic equivalent of task score.

^*^Unless otherwise noted.

**Table 2:** **Summary statistics and Pearson correlation coefficients of PSS scores at each visit**

Visit	n subjects	Mean (SD)	Visit 1	Visit 2	Visit 3	Visit 4
1	987	26.8 (6.8)	1.00	0.44	0.24	0.20
2	699	26.4 (6.7)		1.00	0.32	0.36
3	410	25.3 (7.6)			1.00	0.34
4	148	25.6 (8.2)				1.00

Air pollution and meteorology distributions over the study period are reported in Table 3. Daily concentrations of PM_2.5_ during the study period were generally below the US National Ambient Air Quality Standard (35 μg/m^3^); only 37 of the 4,680 days (0.8%) with PM_2.5_ daily concentration measured exceeded the daily standard. A strong correlation was observed between dailyPM_2.5_ and SO_4_^2-^ concentrations, and fair to moderate correlations were observed between daily PM_2.5_ and BC, and NO_2_ concentrations. PM_2.5_ was weakly inversely correlated with PNC. Fair to moderate correlations were also observed between daily BC and NO_2_, and SO_4_^2-^.

**Table 3:** **Summary statistics and Pearson correlation coefficients of 24-hour mean air pollutant concentrations and meteorological variables**

	Summary statistics			***r***						
	n days	Mean (SD)	Median (IQR)	PM _2.5_	BC	PNC	SO _4_^2-^	NO _2_	O _3_	Apparent temperature
PM_2.5,_ μg/m^3^	4,680	11.0 (6.4)	9.3 (6.6, 13.7)	1.00	0.68	-0.10	0.83	0.49	0.22	0.32
BC_,_ μg/m^3^	4,673	0.9 (0.6)	0.8 (0.5, 1.2)		1.00	0.07	0.49	0.66	-0.13	0.18
PNC, counts per cm^3^	2,580	23,615 (12,569)	20,613 (13,616, 31.548)			1.00	-0.22	0.46	-0.35	-0.67
SO_4_^2-^, μg/m^3^	2,816	3.0 (2.4)	2.3 (1.5, 3.7)				1.00	0.24	0.39	0.41
NO_2_, ppm	4,747	0.02 (0.01)	0.02 (0.01, 0.03)					1.00	-0.23	-0.14
O_3_, ppm	4,740	0.02 (0.01)	0.02 (0.01, 0.03)						1.00	0.47
Apparent temperature, °C	4,734	10.1 (10.2)	8.7 (1.3, 18.9)							1.00

*Abbreviations:* *BC* black carbon, *IQR* interquartile range, *NO*_*2*_ nitrogen dioxide, *O*_*3*_ ozone, *PM*_*2.5*_ fine particulate matter ≤ 2.5 μm in aerodynamic diameter, *PNC* particle number counts, *SD* standard deviation, *SO*_*4*_^*2-*^ sulfate.

A comparison of the distributions and correlations between PM_2.5_ and BC as measured from the stationary monitoring site and estimated from the spatio-temporal predictive models are summarized in Tables S2 and S3, respectively, in Additional file 1. The mean PM_2.5_ concentration at all moving averages were higher according to the predictive model compared with the stationary monitor (Additional file 1: Table S2). Mean PM_2.5_ as measured from the stationary monitor was also strongly correlated with the corresponding mean concentration from the predictive model at all moving averages. In contrast, the mean BC concentration as estimated from the predictive model was considerably lower than the mean concentration measured from the stationary monitor (Additional file 1: Table S3), and the mean BC concentrations from the stationary monitoring site and the predictive model were weakly correlated at all moving averages.

**Figure 1:**
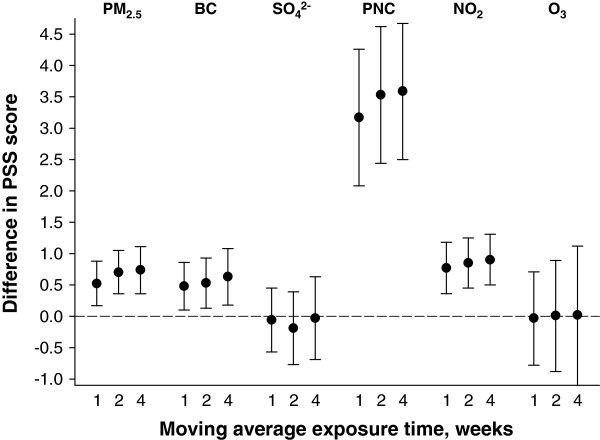
**Adjusted difference in PSS score per interquartile range increase in moving average air pollution exposure measured from stationary monitors.** Associations were estimated in linear mixed effect regression with random intercept for participant after adjustment for seasonality, weekday of visit, 24-hour mean apparent temperature, age, race, years of education, use of anti-depressant medication, and physical activity. Abbreviations: PSS – 14-item Perceived Stress Scale; PM_2.5_ –particulate matter with an aerodynamic diameter of <2.5 μm; BC – black carbon; NO_2_ – nitrogen dioxide; O_3_ – ozone; PNC – particle number counts; SO_4_^2-^ - sulfate.

**Figure 2:**
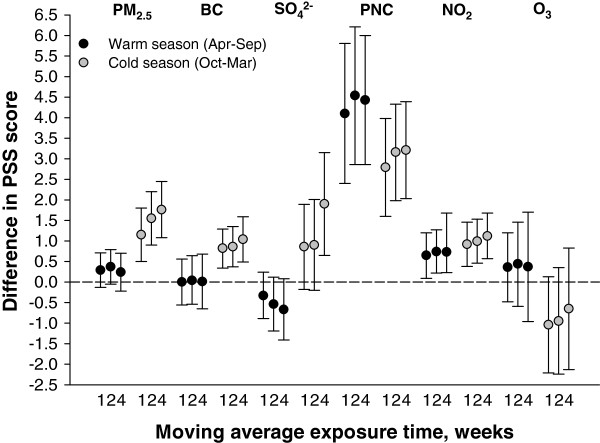
**Adjusted difference in PSS score per interquartile range increase in moving average air pollution exposure measured from stationary monitors in warm and cold seasons.** Associations were estimated in linear mixed effect regression with random intercept for participant after adjustment for seasonality, weekday of visit, 24-hour mean apparent temperature, age, race, years of education, use of anti-depressant medication, and physical activity. Associations for warm (April-September) and cold (October-March) seasons are estimated from interactions between warm/cold season and moving average exposure. Abbreviations: PSS – 14-item Perceived Stress Scale; PM_2.5_ –particulate matter with an aerodynamic diameter of <2.5 μm; BC – black carbon; NO_2_ – nitrogen dioxide; O_3_ – ozone; PNC – particle number counts; SO_4_^2-^ - sulfate.

Statistically significant (p < 0.05) positive associations were observed between medium-term exposures to PM_2.5_, BC, PNC, and NO_2_ measured from the stationary monitoring sites at moving averages of 1, 2, and 4 weeks, and PSS score, such that higher air pollution levels were associated with higher stress rating (Figure 1). After adjustment for all covariates, an interquartile range increase in 1-week average pollutant exposure was associated with a 0.5 point (95% confidence interval [CI]: 0.2, 0.9) increase in PSS score for a 4.7 μg/m^3^ increase in PM_2.5_; a 0.5 point (95% CI: 0.1, 0.9) increase in PSS score for a 0.5 μg/m^3^ increase in BC; and a 0.8 point (95% CI: 0.4, 1.2) increase in PSS score for a 0.006 ppm increase in NO_2_. The strongest associations were observed for PNC; a 15,997 counts/cm^3^ interquartile range increase in PNC was associated with 3.2 point increase in PSS score (95% CI: 2.1, 4.3). Associations for 2 and 4-week moving averages for PM_2.5_, BC, PNC, and NO_2_ were similar compared with associations for the 1-week moving average. All moving averages of SO_4_^2-^ and O_3_ exposures were not associated with PSS score. Observed associations between air pollution and PSS score presented in Figure 2 were largely unchanged after excluding participant-visits with reported anti-depressant medication use (data not shown) and after additional adjustment for smoking status and alcohol consumption (Additional file 1: Table S4). Only slight differences, if at all, were observed for the estimated associations as shown in Figure 1 (and Additional file 1: Table S4) after assigning an inverse probability weight, derived from the probability of being included in the analysis, to the baseline visit (Additional file 1: Table S5). Overall, little difference was observed between weighted models (as shown in Figure 1 and Additional file 1: Table S4) and unweighted models, although the estimated associations were of slightly lower magnitude after inverse probability of censoring weights were incorporated (Additional file 1: Table S6).

Figure 2 summarizes the associations between PSS score and medium-term exposures measured from the stationary monitoring sites by season. Statistically significant interactions were observed between warm/cold season and PM_2.5_, BC, and SO_4_^2-^ at all moving averages; positive associations between PSS score and these pollutants were seen in mainly in colder months. For PNC and NO_2_, positive associations between PSS score and exposure were observed in both warm and cold seasons and the confidence intervals widely overlapped each other. A statistically significant interaction was also observed between warm/cold season and 1-week average O_3_; positive and inverse associations between PSS and moving average O_3_ exposure were observed in warm and cold seasons, respectively. As shown in Figure S2 (Additional file 1), overall there were minimal differences in the estimated associations without adjustment for seasonality (sine and cosine of calendar day) compared to those presented in Figure 2.

Table 4 summarizes the adjusted differences in PSS score and medium-term exposures to predicted PM_2.5_ and BC estimated from the spatio-temporal models. Statistically significant positive associations were observed between PSS score and predicted PM_2.5_ at all moving averages in the pooled model. Consistent with Figure 2, interactions were also present between warm/cold season and predicted PM_2.5_ at all moving averages; the positive associations were of considerably higher magnitude for the cold season. Compared with the associations for PM_2.5_ measured from the stationary monitoring site as presented in Figures 1 and 2, the associations for PM_2.5_ estimated from the spatio-temporal model were generally of slightly higher magnitude with wider variation. Compared with the associations for BC measured from the stationary monitoring site as presented in Figures 1 and 2, a similar pattern of findings were observed for predicted BC estimated from the spatio-temporal model. However, the associations for predicted BC were moderately lower in magnitude; positive associations between PSS score and predicted BC in the pooled model and in the cold season were of marginal statistical significance.

**Table 4:** Adjusted difference in PSS score* per interquartile range increase in moving average PM_**2.5**_ **and BC exposure as predicted from spatio-temporal models**

	PM _2.5_			BC		
	Pooled	Warm season (Apr-Sep)	Cold season (Mar-Oct)	Pooled	Warm season (Apr-Sep)	Cold season (Mar-Oct)
Moving average	β (95% CI)	β (95% CI)	β (95% CI)	β (95% CI)	β (95% CI)	β (95% CI)
1-week	0.80 (0.15, 1.44)	0.43 (-0.43, 1.29)	1.34 (0.38, 2.30)	0.32 (-0.04, 0.68)	0.03 (-0.55, 0.62)	0.48 (0.03, 0.92)
2-week	1.07 (0.43, 1.71)	0.56 (-0.33, 1.46)	1.78 (0.83, 2.74)	0.28 (-0.09, 0.64)	0.04 (-0.53, 0.62)	0.41 (-0.04, 0.86)
4-week	0.96 (0.41, 1.51)	0.31 (-0.47, 1.08)	1.79 (0.93, 2.65)	0.30 (-0.06, 0.67)	0.06 (-0.51, 0.62)	0.46 (-0.00, 0.92)

*Abbreviations:* *PSS* 14-item Perceived Stress Scale, *PM*_*2.5*_ particulate matter with an aerodynamic diameter of <2.5 μm, *BC* black carbon.

^*^As estimated in linear mixed effect regression with random intercept for participant adjusting for seasonality, weekday of visit, 24-hour mean apparent temperature, age, race, years of education, use of anti-depressant medication, and physical activity; associations for warm and cold months are estimated from interactions between warm/cold season and moving average exposure.

## Discussion

In this sample of predominantly white older men, we observed that medium-term exposures to PM_2.5,_ BC, PNC, and NO_2_, as measured from stationary monitoring sites, were associated with higher perceived stress rating. Notably, PNC, BC, and NO_2_ are all traffic pollutants, suggesting that traffic emissions, and particularly fresh ultrafine particles, are the principal source of these associations. Season modified the associations for specific pollutants, and the associations between PM_2.5_, BC, and SO_4_^-2^ and higher PSS scores were observed mainly in colder months, while O_3_ was associated with lower PSS scores during colder months. A consistent pattern of findings were also observed for predicted PM_2.5_ and BC estimated from the spatio-temporal models, although the associations were of higher magnitude for predicted PM_2.5_ and of lower magnitude for predicted BC compared with the associations for these exposures measured from the stationary monitoring site.

While prior work focused on the relation between air pollution levels and perceived air quality [12, 13, 27, 49], this is the first large scale study to investigate objectively measured air pollutant levels in association with non-specific perceived stress, where the range of air pollution levels observed in during the study period were unlikely to alter perceptions of air quality. The observed associations between air pollution and perceived stress may offer insight into recent findings linking air pollution with depressive symptoms and hospitalizations for depression in studies conducted in Korea and Canada [5, 6]. In the former study, investigators identified associations of a 3-day moving average of PM_10_ and O_3_, and 7-day average of NO_2_ with significantly higher reports of depressive symptoms in a cross-sectional community-based study of 537 elderly individuals [6]. Additionally, air pollution was more strongly with emotional symptoms such as feeling less happy than with somatic symptoms. In the latter study, a multi-city time series analysis demonstrated that same-day increases in air concentrations of carbon monoxide, and NO_2_ during warm months, and PM_10_ during cold months were associated with more emergency admissions for depression. If air pollution increases perceptions of stress, and higher levels of stress trigger more depressive symptoms and episodes, this may be one pathway by which air pollution alters depressive status.

That Szyszkowicz and colleagues [5] observed associations for PM_10_ and depression admissions during cold months is also of interest. Season also modified the associations between higher PSS scores and PM_2.5_, BC, and SO_4_^-2^, components of ambient particulate matter, in the present analysis such that these associations were also observed mainly in colder months. How season may affect susceptibility to higher perceived stress rating in association with air pollution in this study population is not clear and merits further investigation. Additionally, while PM_2.5_, BC, and SO_4_^-2^ were associated with higher PSS score during colder months, contrasting findings were observed for O_3_, in which higher exposure was observed with lower PSS score during colder months. The contrast in findings between these pollutants in association with PSS score is not clear, but opposing directions for associations between PM_2.5_ and O_3_ with arterial blood pressure have been observed in a panel study of older adults with diabetes living in the Boston Metropolitan area [50].

The underlying biological mechanisms by which air pollution may actually lead to altered perceptions of stress are as yet unknown. Experimental studies in mice have demonstrated associations between acute and chronic exposures to particulates and activation of the hypothalamic-pituitary-adrenal axis [28] and hippocampal pro-inflammatory cytokine expression [3], respectively. The former study observed that 4-hour exposure to particles increased plasma levels of adrenocorticotropic hormone and the glucocorticoid corticosterone, with a corresponding increase in markers of glucocorticoid activity [28]. Lead, a ubiquitous environmental pollutant known for its neurotoxic effects, has also been observed to be associated with rat hippocampal pro-inflammatory cytokine expression [51]. Findings from experimental and observational studies also suggest that increased glucocorticoid activity study may be associated with environmental reactivity [52], perceived stress [53], and stress-related disorders [54]. Taking these findings together, we hypothesize that the association between ambient air pollution and higher perceived stress may be mediated by brain inflammation and glucocorticoid activity.

The public health relevance of the present findings are multifold, considering that perceived stress is a risk factor for affective psychiatric disorders [15–17], inflammation [18, 19], and cardiovascular disease and mortality [20–23]. While previous studies show that psychological stress may enhance susceptibility for air-pollution-related health effects [55–57], the current findings suggest that stress may also be a mediating factor which should be taken into consideration. Thus, it may be hypothesized that the physical environment may alter mental health in addition to physical health, and possibly affect physical health by first altering mental health.

This study has a number of strengths including prospective design to investigate the role of air pollution from various sources on repeated measures of perceived stress, use of validated spatio-temporal models for assessment of exposure to air pollution, methods to address selection bias, and adjustment for multiple confounders. However, there are several limitations should be considered. While observed associations are not confounded by such factors as age, individual level indicators of socioeconomic status, psychiatric medication use, physical activity, temperature, and seasonality which could influence air pollution exposure as well as levels of perceived stress, there may be unmeasured confounding from other factors associated with perceived stress in this study sample such as environmental noise. Road traffic and aircraft noise have also been shown to be associated with higher salivary cortisol levels [58, 59] and symptoms of anxiety and perceived stress [60–62]. For this analysis, we hypothesize that the factors (i.e. meteorology, wind speed, wind direction) that explain the week to week variation in exposure to traffic-related air pollution do not strongly overlap with those that explain the week to week variation in exposure to traffic-related noise. Moreover, while levels of pollution were relatively low it is as yet unclear at what levels individuals can detect changes. Since perception of air quality itself may also correlate with actual exposure levels, the observed effects from this study could be due in part to subjective perceptions of pollution; however, it has also been shown perceived air quality may actually be a function of mood states and anxiety [63, 64]. In fact, PM_2.5_ daily concentration measured at the central monitoring site exceeded the daily standard in only less than 1% of all days in the study period, thus it is unlikely that perceived air quality would have more than minimal influence on the observed associations in this study.

Exposure measurement error is also a potential source of bias as we relied on central monitoring site for measurement of ambient particulate concentrations, which may not be representative of where these participants reside. The interpretation of the overall findings for predicted PM_2.5_ and BC estimated from the spatio-temporal models was generally consistent with PM_2.5_ and BC measured from the stationary monitoring site; however the associations for predicted PM_2.5_ and BC were of higher and lower magnitude, respectively, in comparison with the associations for their respective exposures measured from the stationary monitoring site. The comparison of associations between PM_2.5_ and PSS scores utilizing stationary monitoring site measurements and spatio-temporal predictive estimates suggests that there may be a higher degree of non-differential exposure measurement error from using the stationary monitoring site exposure measurements leading to a bias towards the null. However, spatial smoothing induces Berskson error, and the marginally wider confidence intervals observed for the spatio-temporal predictive estimates of PM_2.5_ are consistent with that. In contrast, the estimated associations were of lower magnitude utilizing the spatio-temporal predictive estimates of BC compared with the stationary site measurements of BC while the confidence intervals were of similar length. PM_2.5_ is generally considered to be a regional pollutant and BC is a near-roadway pollutant (mobile sources), and BC generally has more measurement error in regional models [65]. In this study, the spatial R^2^ of the land-use regression BC model is much lower than the satellite-derived aerosol optical depth PM_2.5_ model; thus there may be more classical measurement error in the spatial variation of BC exposure. Lastly, the population in this analysis is relatively homogeneous, consisting of predominantly white older men. Thus, these results cannot be generalized to other populations without further research on how effects vary by age, gender and race.

## Conclusions

In conclusion, we identified novel associations between medium-term exposures to air pollution and higher stress rating in this sample of predominantly white elderly men, more so in colder months for specific pollutants. The findings support the hypothesis that perceived stress may be a mediating factor for the association between air pollution and affective disorders.

## Electronic supplementary material

Additional file 1: **Supplementary material.** (DOC 192 KB)

## Abbreviations

PSS: 14-item Perceived stress scale
NAS: VA normative aging study
PM_2.5_: Particulate matter ≤ 2.5 μm in diameter
BC: Black carbon
PNC: Particle number counts
SO_4_^2-^: Sulfate
O_3_: Ozone
NO_2_: Nitrogen dioxide.
