# Supplementary material for: Associations between air pollution and perceived stress: the Veterans Administration Normative Aging Study
Source: Environ Health. 2015 Jan 27;14:10. doi: 10.1186/1476-069X-14-10 (PMC4417295; doi:10.1186/1476-069X-14-10)
Supplement: Supplementary file 1 — Additional file 1: Supplementary material. (DOC 192 KB) [file 12940_2014_846_MOESM1_ESM.doc]

ADDITIONAL FILE 1

SUPPLEMENTARY MATERIAL

METHODS

*Air pollution and meteorology data*

Three sites (Boston/Harrison ave., Boston/Kenmore Sq, Lynn) were used for estimating the mean NO_2_ exposure, and 2 sites (Boston/Harrison ave., Lynn) were used for estimating the mean O_3_ exposure. The Boston/Harrison ave. site is expected to characterize urban background population exposure. The Boston/Kenmore Sq site is near a 5 road intersection and is expected to characterize the highest exposure concentration. The Lynn site is a Photochemical Assessment Monitoring Station (PAMS) designed to measure ozone precursors and meteorological parameters in order to provide data about ozone formation and the effect of precursor controls on ozone production; specifically, Lynn is a Type 2 PAMS site that is at or near the downwind edge of the urban area. Because the mean concentrations can be influenced by the specific monitors that contribute data, we used a method to calculate means that was independent of the monitors and therefore did not introduce changes in mean exposure estimates from day to day (E1). The annual mean for each pollutant was calculated for each monitor for each year. These monitor- and year-specific means were subtracted from the hourly measurement for each monitor, and the difference was divided by the monitor-specific standard deviation to produce a standardized deviation. The standardized deviations for all reporting monitors were averaged for each hour. The average standardized deviation was then multiplied by the standard deviation of all the centered measurements for the year and added to the annual average of all the monitors to produce an average concentration for each hour.

*Assessment of predicted black carbon*

BC exposures were estimated from a spatiotemporal model that we developed and validated previously, which has been described in detail previously (Gryparis et al. 2007). Daily concentrations at the Boston central-site monitor were used as a predictor to reflect average concentration levels for a given day, serving as a direct estimate of the daily time effect. Data from 82 other stationary air monitors were used to fit the model and estimate the effect of each covariate in the land-use regression model. Covariates in the BC prediction model included measures of land use for each address (cumulative traffic density within 100 m, population density, distance to nearest major roadway and per cent urbanisation), geographic information system, location (latitude and longitude), daily meteorological factors (apparent temperature, wind speed and height of the planetary boundary layer) and temporal factors (day of week and day of season).

Separate models were fit for the warm and cold season. Interaction terms between the

temporal meteorological predictors and land-use variables allowed for space–time interactions. Regression splines allowed main effect terms to non-linearly predict exposure levels, and thin-plate splines modelled the residual spatial variability additional spatial variability unaccounted for by the spatial predictors. A latent variable framework was used to integrate BC and EC exposure data, where BC and EC measurements were treated as surrogates of some true unobservable traffic exposure variable, see Gryparis et al. for further details.

Our BC model showed more than a threefold range of variation in long-term average exposure across the measuring sites, and the adjusted R^2^ for this model was 0.83. A subsequent validation sample using monthly monitoring data collected at 30 additional locations found an average correlation of 0.59 between predicted values and observed BC levels. All addresses of participants in the NAS have been geocoded and we used the model to generate daily predicted BC at the address of each participant. Daily BC predictions outside of the observed range of the monitored exposure measurements were excluded.

*Assessment of predicted PM_2.5_*

Predicted PM_2.5_ at the residence for the years 2000 through 2007 were generated by a novel exposure model developed recently by Kloog et al. (2013) for assessing temporally and spatially resolved PM_2.5_ exposures for epidemiological studies. This new method uses MODIS (Moderate Resolution Imaging Spectroradiometer) satellite-derived AOD measurements to predict daily PM_2.5_ concentration levels at a 10 × 10 km spatial resolution in New England beginning in the year 2000 based on daily physical measurements of a surrogate for PM_2.5_ concentrations in each grid cell. In brief, we performed day-specific calibrations of AOD data using ground PM_2.5_ measurements from 78 monitoring stations and land use regression and meteorological variables (temperature, wind speed, visibility, elevation, distance to major roads, percent of open space, point emissions, and area emissions). To estimate PM_2.5_ concentrations in each grid cell on each day we calibrated the AOD– PM_2.5_ relationship for each day using data from grid cells with both monitor and AOD values, using mixed models with random slopes for day and nested regions. In a second stage, we estimated exposures on days when AOD measures were not available (due to cloud coverage or snow, for example). A model was fit with a smooth function of latitude and longitude and a random intercept for each cell (similar to universal kriging) that takes advantage of associations between grid cell AOD values and PM_2.5_ data from monitors located elsewhere and associations with available AOD values in neighboring grid cells.

To validate our model, the data set was repeatedly randomly divided into 90% and 10% splits. Predictions for the held-out 10% of the data were made from the model fit of the remaining 90% of the data. This “out of sample” process was repeated 10 times and cross-validated R^2^ values were computed. The first stage calibrations resulted in high out-of-sample 10-fold cross-validated R^2^ (mean out-of-sample R^2^ = 0.85). Even for location-day combinations without AOD data (the second stage models), our model performance was excellent (mean out-of-sample R^2^ = 0.81). Importantly, these R^2^ are for daily observations. To check for bias we regressed the measured PM_2.5_ values against the predicted values in each site on each day.

REFERENCES

1. Gryparis A, Coull B, Schwartz J, et al. Semiparametric latent variable regression models for spatiotemporal modelling of mobile source particles in the greater Boston area. J R Stat Soc Ser C 2007;56:183–209.

2. Kloog I, Ridgway B, Koutrakis P, Coull BA, Schwartz JD. Long- and short-term exposure to PM2.5 and mortality: using novel exposure models. Epidemiology 2013;24:555-61.

Abbreviations: PSS-14 – 14-item Perceived Stress Scale; PM_2.5_ –particulate matter with an aerodynamic diameter of < 2.5 μm; BC – black carbon; NO_2_ – nitrogen dioxide; O_3_ – ozone; PNC – particle number concentration; SO_4_^2-^ - sulfate.

**Figure S1** Flowchart of study participants in VA Normative Aging Study 1995-2007

**Table S1** Distribution of individual characteristics between participants and non-participants at the first visit^*^: VA Normative Aging Study 1995-2007

|  | **Participants (n=987)** | **Non-participants (n=131)** | **P-value^**^** |
| --- | --- | --- | --- |
| Age, mean (SD) | 69.1 (7.0) | 73.3 (8.6) | **<0.0001** |
| Body mass index, mean (SD) | 28.0 (3.8) | 27.2 (4.1) | 0.06 |
| Race |  |  |  |
| White | 972 (98) | 127 (97) | 0.27 |
| Black | 15 (2) | 4 (3) |
| Years of education, mean (SD) | 14.8 (2.9) | 13.8 (2.8) | **<0.01** |
| Missing information on education, n (%) | 0 | 49 (37) | N/A |
| Physical activity (METS/week), mean (SD) | 17.0 (12.0) | 15.7 (27.1) | 0.46 |
| Missing information on physical activity, n (%) | 25 (3) | 25 (19) | N/A |
| Coronary heart disease, n (%) | 254 (26) | 39 (30) | 0.34 |
| Diabetes mellitus, n (%) | 142 (14) | 26 (20) | 0.12 |
| Hypertension, n (%) | 655 (66) | 93 (71) | 0.32 |
| Smoking status, n (%) |  |  |  |
| Current | 57 (6) | 15 (12) | **0.02** |
| Former | 656 (66) | 84 (64) | 0.62 |
| Never | 713 (72) | 99 (76) | 0.47 |
| ≥ 2 drinks / day, n (%) | 215 (22) | 20 (15) | 0.09 |

^*^ Median year of first visit in study period for participants and non-participants was 1995

^**^ T-test and Chi-square tests for were used for comparing means and proportions between groups, respectively.

**Table S2** Summary statistics and Pearson Correlation Coefficients of moving average PM_2.5_ measured from the central site monitor (CS) and estimated from the spatio-temporal predictive model (P) for all person-visits included in the analysis (n=902)

|  | **Summary Statistics** | | ***r*** | | | | | |
| --- | --- | --- | --- | --- | --- | --- | --- | --- |
|  | **Mean (SD)** | **Median (IQR)** | **7-day PM_2.5_, CS, μg/m^3^** | **14-day PM_2.5_, CS, μg/m^3^** | **28-day PM_2.5_, CS, μg/m^3^** | **7-day PM_2.5_, P, μg/m^3^** | **14-day PM_2.5_, P, μg/m^3^** | **28-day PM_2.5_, P, μg/m^3^** |
| **7-day PM_2.5_, CS, μg/m^3^** | 10.1 (3.3) | 9.8 (7.7, 12.0) | 1.00 | 0.81 | 0.67 | 0.79 | 0.65 | 0.54 |
| **14-day PM_2.5_, CS, μg/m^3^** | 10.2 (2.9) | 9.9 (8.0, 11.7) |  | 1.00 | 0.86 | 0.62 | 0.77 | 0.69 |
| **28-day PM_2.5_, CS, μg/m^3^** | 10.3 (2.8) | 10.0 (8.5, 11.8) |  |  | 1.00 | 0.49 | 0.65 | 0.80 |
| **7-day PM_2.5_, P, μg/m^3^** | 10.9 (3.2) | 10.4 (8.5, 12.6) |  |  |  | 1.00 | 0.82 | 0.63 |
| **14-day PM_2.5_, P, μg/m^3^** | 10.9 (2.8) | 10.5 (8.9, 12.6) |  |  |  |  | 1.00 | 0.83 |
| **28-day PM_2.5_, P, μg/m^3^** | 11.1 (2.5) | 10.7 (9.3, 12.2) |  |  |  |  |  | 1.00 |

**Table S3** Summary statistics and Pearson Correlation Coefficients of moving average BC measured from the central site monitor (CS) and estimated from the spatio-temporal predictive model (P) for all person-visits included in the analysis (n=1,874)

|  | **Summary Statistics** | | ***r*** | | | | | |
| --- | --- | --- | --- | --- | --- | --- | --- | --- |
|  | **Mean (SD)** | **Median (IQR)** | **7-day BC, CS, μg/m^3^** | **14-day BC, CS, μg/m^3^** | **28-day BC, CS, μg/m^3^** | **7-day BC, P, μg/m^3^** | **14-day BC, P, μg/m^3^** | **28-day BC, P, μg/m^3^** |
| **7-day BC, CS, μg/m^3^** | 1.0 (0.4) | 0.9 (0.7, 1.2) | 1.00 | 0.91 | 0.85 | 0.25 | 0.19 | 0.14 |
| **14-day BC, CS, μg/m^3^** | 1.0 (0.4) | 1.0 (0.7, 1.2) |  | 1.00 | 0.94 | 0.19 | 0.20 | 0.14 |
| **28-day BC, CS, μg/m^3^** | 1.0 (0.4) | 1.0 (0.8, 1.3) |  |  | 1.00 | 0.17 | 0.18 | 0.18 |
| **7-day BC, P, μg/m^3^** | 0.5 (0.3) | 0.5 (0.3, 0.7) |  |  |  | 1.00 | 0.97 | 0.93 |
| **14-day BC, P, μg/m^3^** | 0.5 (0.3) | 0.5 (0.3, 0.7) |  |  |  |  | 1.00 | 0.97 |
| **28-day BC, P, μg/m^3^** | 0.5 (0.3) | 0.5 (0.3, 0.7) |  |  |  |  |  | 1.00 |

**Table S4** Adjusted differences in PSS Score per interquartile range increases in air pollution averaged over 1, 2, and 4 weeks

|  | **1-week** | **2-week** | **4-week** |
| --- | --- | --- | --- |
|  | **β (95%CI)** | **β (95%CI)** | **β (95%CI)** |
| PM_2.5_, μg/m^3^ |  |  |  |
| Minimally adjusted | 0.50 (0.15, 0.86) | 0.68 (0.33, 1.03) | 0.71 (0.33, 1.08) |
| Fully adjusted | 0.52 (0.17, 0.88) | 0.70 (0.36, 1.05) | 0.74 (0.36, 1.11) |
| Fully adjusted + smoking, alcohol | 0.52 (0.17, 0.88) | 0.70 (0.36, 1.05) | 0.74 (0.36, 1.11) |
| BC, μg/m^3^ |  |  |  |
| Minimally adjusted | 0.46 (0.08, 0.84) | 0.50 (0.11, 0.90) | 0.59 (0.14, 1.03) |
| Fully adjusted | 0.48 (0.10, 0.86) | 0.53 (0.13, 0.93) | 0.63 (0.18, 1.08) |
| Fully adjusted + smoking, alcohol | 0.48 (0.10, 0.86) | 0.53 (0.13, 0.92) | 0.63 (0.18, 1.08) |
| PNC, counts per cm^3^ |  |  |  |
| Minimally adjusted | 3.03 (1.94, 4.12) | 3.39 (2.31, 4.48) | 3.41 (2.33, 4.50) |
| Fully adjusted | 3.17 (2.08, 4.26) | 3.53 (2.44, 4.62) | 3.59 (2.50, 4.67) |
| Fully adjusted + smoking, alcohol | 3.19 (2.10, 4.29) | 3.58 (2.49, 4.68) | 3.62 (2.53, 4.71) |
| SO_4_^2-^, μg/m^3^ |  |  |  |
| Minimally adjusted | -0.03 (-0.55, 0.48) | -0.18 (-0.76, 0.40) | 0.03 (-0.64, 0.70) |
| Fully adjusted | -0.06 (-0.57, 0.45) | -0.19 (-0.77, 0.39) | -0.03 (-0.69, 0.63) |
| Fully adjusted + smoking, alcohol | -0.02 (-0.53, 0.49) | -0.15 (-0.73, 0.43) | -0.00 (-0.67, 0.66) |
| NO_2_, ppm |  |  |  |
| Minimally adjusted | 0.75 (0.34, 1.16) | 0.82 (0.42, 1.22) | 0.85 (0.45, 1.25) |
| Fully adjusted | 0.77 (0.36, 1.18) | 0.85 (0.45, 1.25) | 0.90 (0.50, 1.31) |
| Fully adjusted + smoking, alcohol | 0.77 (0.36, 1.18) | 0.85 (0.45, 1.25) | 0.90 (0.50, 1.30) |
| O_3_, ppm |  |  |  |
| Minimally adjusted | -0.10 (-0.84, 0.65) | -0.05 (-0.94 (0.84) | -0.02 (-1.12, 1.09) |
| Fully adjusted | -0.03 (-0.78, 0.71) | 0.01 (-0.88, 0.89) | 0.02 (-1.18, 1.12) |
| Fully adjusted + smoking, alcohol | -0.04 (-0.78, 0.71) | 0.00 (-0.88, 0.89) | 0.02 (-1.08, 1.12) |

Adjusted differences^*^ in PSS score in association with interquartile range increases in 1, 2, and 4 week moving average of exposure measured from area monitoring sites, as estimated in linear mixed effect regression with random intercept for participant. Minimally adjusted model includes adjustment for seasonality, weekday of visit, 24-hour mean apparent temperature and age; fully adjusted model includes additional adjustment for race, years of education, use of anti-depressant medication, and physical activity; additional adjustment for smoking status (current former, never as reference) and alcohol consumption (≥ 2 drinks/day, < 2 drinks/day as reference) as sensitivity analysis. Abbreviations: PSS – 14-item Perceived Stress Scale; PM_2.5_ –particulate matter with an aerodynamic diameter of < 2.5 μm; BC – black carbon; NO_2_ – nitrogen dioxide; O_3_ – ozone; PNC – particle number counts; SO_4_^2-^ - sulfate.

**Table S5** Adjusted differences in PSS Score per interquartile range increases in air pollution averaged over 1, 2, and 4 weeks with and without inverse probability weighting at 1^st^ visit

|  | **1-week** | **2-week** | **4-week** |
| --- | --- | --- | --- |
|  | **β (95%CI)** | **β (95%CI)** | **β (95%CI)** |
| PM_2.5_, μg/m^3^ |  |  |  |
| Fully adjusted (Table S4) | 0.52 (0.17, 0.88) | 0.70 (0.36, 1.05) | 0.74 (0.36, 1.11) |
| Fully adjusted w/ IPW at 1^st^ visit | 0.52 (0.17, 0.87) | 0.71 (0.37, 1.06) | 0.74 (0.37, 1.12) |
| BC, μg/m^3^ |  |  |  |
| Fully adjusted (Table S4) | 0.48 (0.10, 0.86) | 0.53 (0.13, 0.93) | 0.63 (0.18, 1.08) |
| Fully adjusted w/ IPW at 1^st^ visit | 0.48 (0.10, 0.86) | 0.54 (0.15, 0.94) | 0.64 (0.20, 1.09) |
| NO_2_, ppm |  |  |  |
| Fully adjusted (Table S4) | 0.77 (0.36, 1.18) | 0.85 (0.45, 1.25) | 0.90 (0.50, 1.31) |
| Fully adjusted w/ IPW at 1^st^ visit | 0.76 (0.35, 1.17) | 0.85 (0.45, 1.25) | 0.90 (0.50, 1.30) |
| O_3_, ppm |  |  |  |
| Fully adjusted (Table S4) | -0.03 (-0.78, 0.71) | 0.01 (-0.88, 0.89) | 0.02 (-1.18, 1.12) |
| Fully adjusted w/ IPW at 1^st^ visit | 0.01 (-0.73, 0.76) | 0.09 (-0.80, 0.98) | 0.11 (-1.00, 1.21) |

Adjusted differences^*^ in PSS score in association with interquartile range increases in 1, 2, and 4 week moving average of exposure measured from area monitoring sites, as estimated in linear mixed effect regression with random intercept for participant. Minimally adjusted model includes adjustment for seasonality, weekday of visit, 24-hour mean apparent temperature and age; fully adjusted model includes additional adjustment for race, years of education, use of anti-depressant medication, and physical activity; additional adjustment for smoking status (current former, never as reference) and alcohol consumption (≥ 2 drinks/day, < 2 drinks/day as reference) as sensitivity analysis. Abbreviations: PSS – 14-item Perceived Stress Scale; PM_2.5_ –particulate matter with an aerodynamic diameter of < 2.5 μm; BC – black carbon; NO_2_ – nitrogen dioxide; O_3_ – ozone.

**Table S6** Adjusted differences in PSS Score per interquartile range increases in air pollution averaged over 1, 2, and 4 weeks with and without inverse probability weighting in subsequent visits

|  | **1-week** | **2-week** | **4-week** |
| --- | --- | --- | --- |
|  | **β (95%CI)** | **Β (95%CI)** | **β (95%CI)** |
| PM_2.5_, μg/m^3^ |  |  |  |
| Fully adjusted (from Table S4) | 0.52 (0.17, 0.88) | 0.70 (0.36, 1.05) | 0.74 (0.36, 1.11) |
| Fully adjusted w/o IPW | 0.60 (0.24, 0.94) | 0.77 (0.42, 1.12) | 0.83 (0.46, 1.20) |
| BC, μg/m^3^ |  |  |  |
| Fully adjusted (from Table S4) | 0.48 (0.10, 0.86) | 0.53 (0.13, 0.93) | 0.63 (0.18, 1.08) |
| Fully adjusted w/o IPW | 0.49 (0.12, 0.87) | 0.56 (0.17, 0.96) | 0.66 (0.22, 1.12) |
| PNC, counts per cm^3^ |  |  |  |
| Fully adjusted (from Table S4) | 3.17 (2.08, 4.26) | 3.53 (2.44, 4.62) | 3.59 (2.50, 4.67) |
| Fully adjusted w/o IPW | 3.17 (2.08, 4.27) | 3.57 (2.48, 4.66) | 3.60 (2.51, 4.68) |
| SO_4_^2-^, μg/m^3^ |  |  |  |
| Fully adjusted (from Table S4) | -0.06 (-0.57, 0.45) | -0.19 (-0.77, 0.39) | -0.03 (-0.69, 0.63) |
| Fully adjusted w/o IPW | -0.03 (-0.55, 0.49) | -0.14 (-0.72, 0.45) | 0.00 (-0.67, 0.68) |
| NO_2_, ppm |  |  |  |
| Fully adjusted (from Table S4) | 0.77 (0.36, 1.18) | 0.85 (0.45, 1.25) | 0.90 (0.50, 1.31) |
| Fully adjusted w/o IPW | 0.79 (0.38, 1.20) | 0.88 (0.48, 1.28) | 0.93 (0.53, 1.32) |
| O_3_, ppm |  |  |  |
| Fully adjusted (from Table S4) | -0.03 (-0.78, 0.71) | 0.01 (-0.88, 0.89) | 0.02 (-1.18, 1.12) |
| Fully adjusted w/o IPW | -0.02 (-0.76, 0.72) | 0.10 (-0.79, 0.99) | 0.19 (-0.91, 1.29) |

Adjusted differences^*^ in PSS score in association with interquartile range increases in 1, 2, and 4 week moving average of exposure measured from area monitoring sites, as estimated in linear mixed effect regression with random intercept for participant. Minimally adjusted model includes adjustment for seasonality, weekday of visit, 24-hour mean apparent temperature and age; fully adjusted model includes additional adjustment for race, years of education, use of anti-depressant medication, and physical activity; additional adjustment for smoking status (current former, never as reference) and alcohol consumption (≥ 2 drinks/day, < 2 drinks/day as reference) as sensitivity analysis. Abbreviations: PSS – 14-item Perceived Stress Scale; PM_2.5_ –particulate matter with an aerodynamic diameter of < 2.5 μm; BC – black carbon; NO_2_ – nitrogen dioxide; O_3_ – ozone; PNC – particle number counts; SO_4_^2-^ - sulfate.

**Figure S2** Adjusted difference in PSS score per interquartile range increase in moving average air pollution exposure measured from stationary monitors in warm and cold seasons without adjustment for sine and cosine of calendar day. Associations were estimated in linear mixed effect regression with random intercept for participant after adjustment for weekday of visit, 24-hour mean apparent temperature, age, race, years of education, use of anti-depressant medication, and physical activity. Associations for warm (April-September) and cold (October-March) seasons are estimated from interactions between warm/cold season and moving average exposure. Abbreviations: PSS – 14-item Perceived Stress Scale; PM_2.5_ –particulate matter with an aerodynamic diameter of < 2.5 μm; BC – black carbon; NO_2_ – nitrogen dioxide; O_3_ – ozone; PNC – particle number counts; SO_4_^2-^ - sulfate.
